# Serum diamine oxidase (DAO) activity as a diagnostic test for histamine intolerance

**DOI:** 10.1186/2045-7022-1-S1-P115

**Published:** 2011-08-12

**Authors:** Ema Music, Mira Silar, Peter Korosec, Mitja Kosnik, Matija Rijavec

**Affiliations:** 1 University Clinic of Respiratory and Allergic Diseases Golnik, Golnik, Slovenia

## 

Histamine intolerance is mainly caused by an imbalance of histamine intake and the capacity for histamine metabolism and degradation. The main enzyme for metabolism of ingested histamine is diamine oxidase (DAO). Determination of DAO activity in serum might be useful for differential diagnosis of histamine intolerance. Over the 3.5-year-long period we have recruited 316 patients with suspicion of histamine intolerance and excluded food allergy together with 20 healthy controls. Serum DAO activity was measured with Enzyme immunoassay for the quantitative determination of histamine-degradation activity by DAO in serum. Twenty patients with histamine intolerance and highly reduced initial activity of serum DAO (<40 HDU/ml) went to a histamine-free diet and after 6 to 12 months of histamine-free diet all clinical parameters and serum for determination of DAO activity were taken again.

We found that DAO activity was significantly lower in patients than in healthy control subjects (P<0.0001). Furthermore, 54 patients had highly reduced activity of DAO (<40 HDU/ml). The main symptoms involved the skin, gastrointestinal tract, respiratory system and eyes. In all 20 patients after the histamine-free diet the main clinical symptoms typical for histamine intolerance have disappeared. Furthermore, the measured values for activity of serum DAO have increased significantly (p<0.0001).

We can conclude that determination of DAO activity in serum is a useful diagnostic tool, together with detailed history to differentiate between food allergy and histamine intolerance. It should be performed in suspected patients with symptoms like headache, tachycardia, urticaria, pruritus, diarrhea and hypotension, where food allergy was excluded. Furthermore, our results showed the benefit of histamine-free diet, since after the diet majority of histamine related symptoms have disappeared as well as the DAO activity in serum has increased.

